# Supplementary material for: Intracellular chromobody delivery by mesoporous silica nanoparticles for antigen targeting and visualization in real time
Source: Sci Rep. 2016 May 13;6:25019. doi: 10.1038/srep25019 (PMC4865863; doi:10.1038/srep25019)
Supplement: Supplementary Information [file srep25019-s1.pdf]

Supplementary Data

Intracellular chromobody delivery by mesoporous silica nanoparticles for antigen targeting and visualization in real time

Hsin-Yi Chiu[a], Wen Deng[b], Hanna Engelke[a], Jonas Helma[b], Heinrich Leonhardt[b,]*, and Thomas Bein[a,]*

[a] Department of Chemistry and Center for NanoScience (CeNS), University of Munich (LMU), Butenandtstrasse 5-13 (E), 81377 Munich, Germany

[b] Department of Biology II and Center for NanoScience (CeNS), University of Munich (LMU), Grosshadernerstrasse 2, 82152 Planegg-Martinsried, Germany

Supplementary Figures

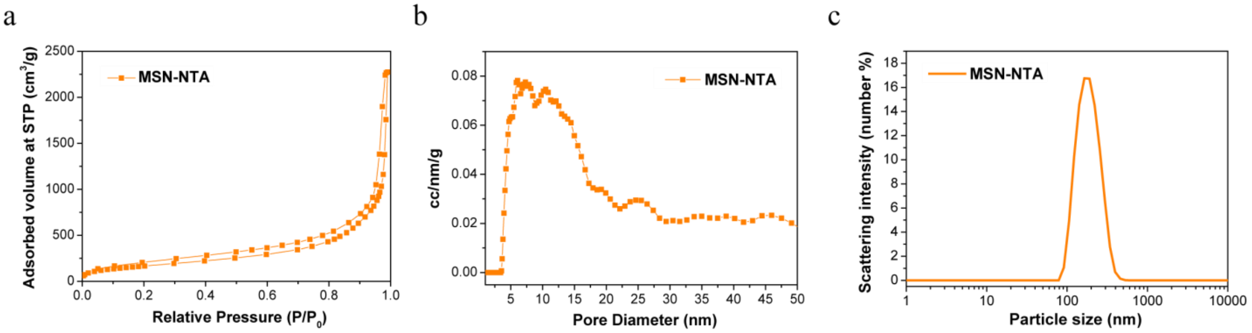

Supplementary Figure 1. Characterization of NTA-conjugated MSNs (MSN-NTA). (a) Nitrogen sorption isotherm, (b) pore size distribution and (c) DLS analysis (particles suspended in EtOH) indicated that MSN-NTA preserved the large-pore mesoporosity and colloidal stability after surface modification.

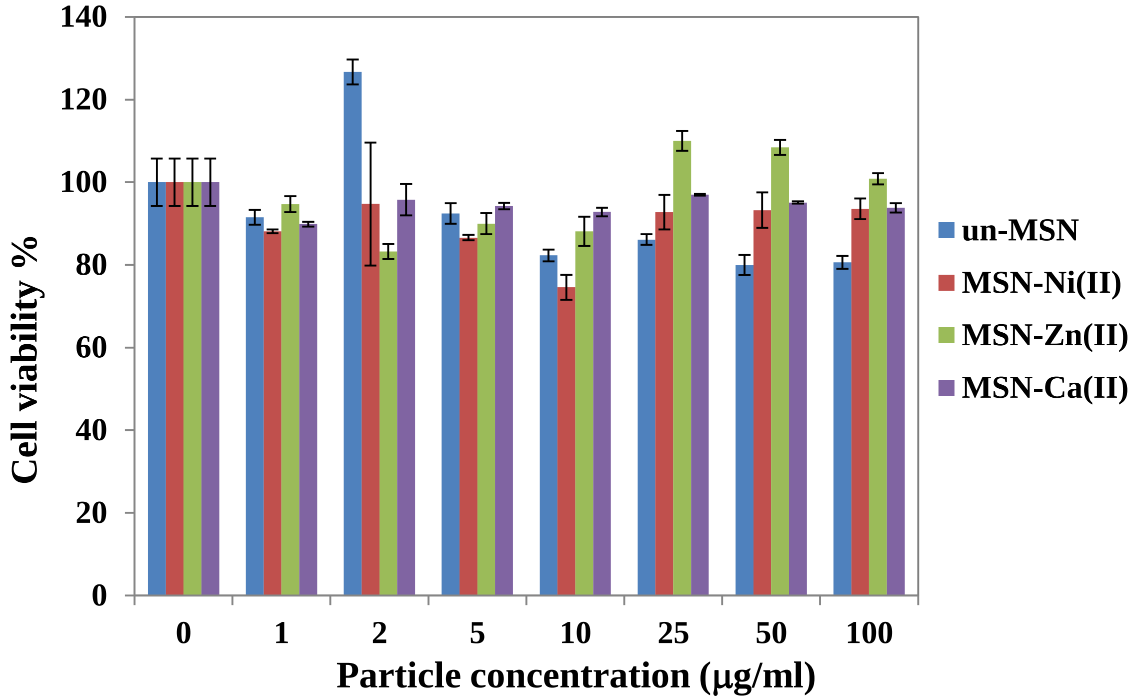

Supplementary Figure 2. Cytotoxicity tests (MTT assay) of different metal-treated MSN-NTA samples (MSN-Ni^{2+}, MSN-Zn^{2+} and MSN-Ca^{2+}) and un-functionalized MSN (un-MSN) on MEF cells (wild type). All the particle types show no significant cytotoxicity below the particle concentration of 100 µg ml^{-1}.

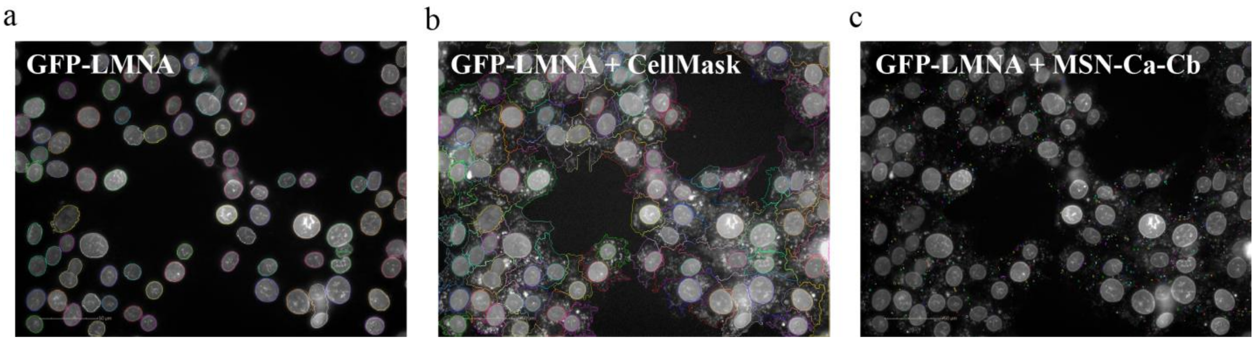

Supplementary Figure 3. High-throughput imaging evaluation of cellular uptake of MSN-Ca-Cbs. MEF-G-LMNA living cells were stained with CellMask orange and imaged at indicated time points after the addition of MSN-Ca-Cbs. Fluorescence from GFP, CellMask orange, and ATTO 647N (labeled on chromobodies) were recorded separately with Operetta high content image analysis system using standard filter for 488 nm, 546 nm and 647 nm emission. Images were analyzed with Harmony® analysis software as the following sequence. (a) The nuclear region is segmented from the background according to GFP-LMNA signal where the GFP-LMNA signal is shown in grey and the circular color lines stand for the segmentation results. Most of the recognized nuclear region fit the GFP-LMNA signal, which indicates a correct segmentation. (b) The cytoplasm region was recognized *via* CellMask signal segmentation. The cytoplasm region is obtained by subtraction of nuclear region obtained from step (a). The border of the segmented cell region was shown as closed color lines around the nuclear region. (c) Recognition of MSN-Ca-Cbs taken up by the cells. Internalized MSN-Ca-Cbs were visualized as spots within the cytoplasm region, and the spots recognition was presented as color dots in the image. The segmented results (nuclei, cytoplasm and MSN-Ca-Cb spots) were used to define populations. Population 1: Cell = GFP-LMNA nucleus plus its surrounding cytoplasm region. Population 2: MSN-uptake cell = cell with more than two MSN-Ca-Cb spots in its cytoplasm and nucleus region. The evaluation results were based on the calculation: (i) % cell take up MSN-Ca-

Cbs = MSN-uptake cells/total cells; (ii) average MSN-Ca-Cb spots per MSN-uptake cell = total spots number in all MSN-uptake cells/MSN-uptake cells.

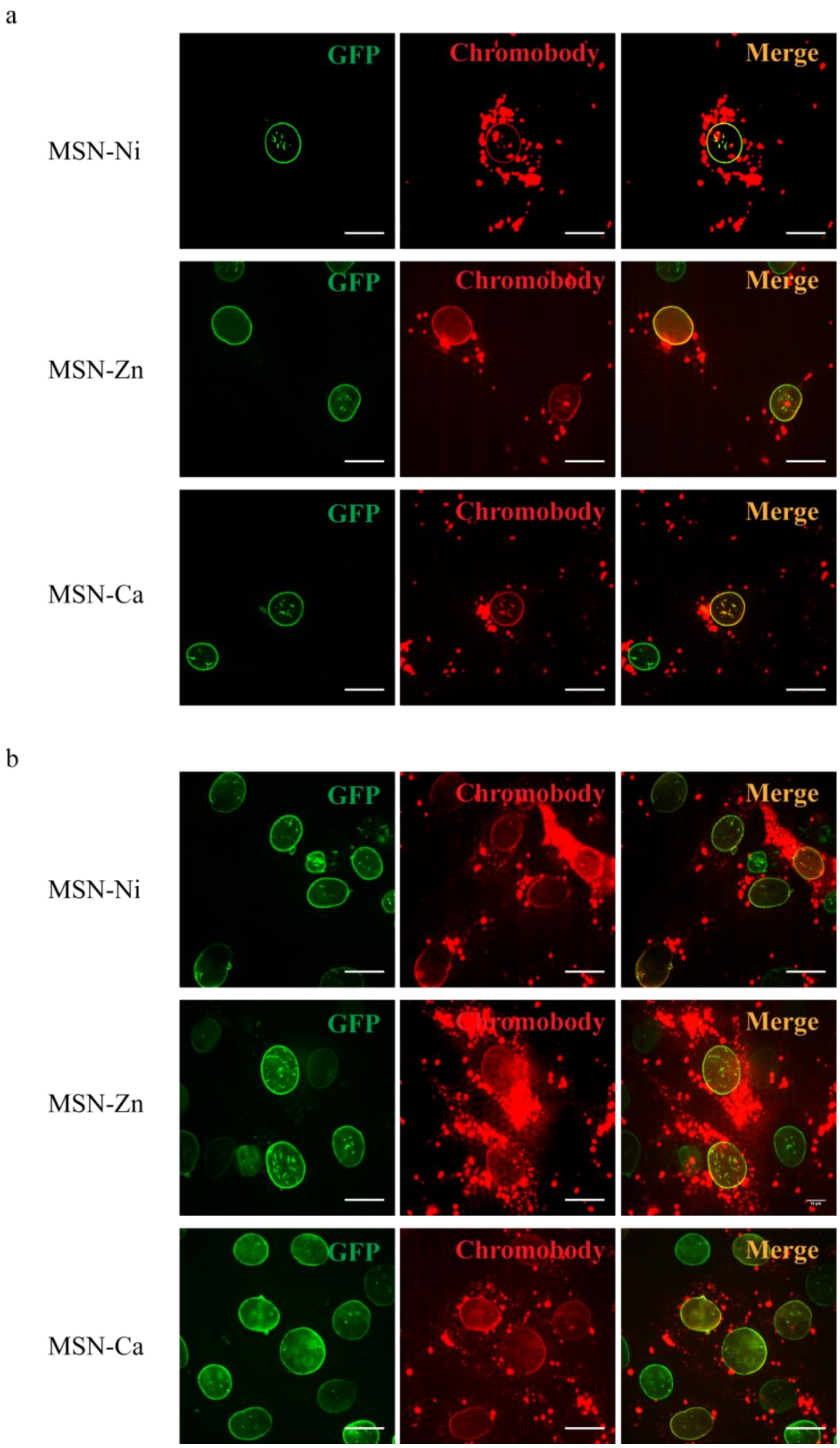

Supplementary Figure 4. Intracellular delivery of chromobodies *via* different metal-treated MSNs. (a) 4 h after MSN-Ni-Cb/MSN-Zn-Cb/MSN-Ca-Cb incubation. (b) 96 h after MSN-Ni-Cb/MSN-Zn-Cb/MSN-Ca-Cb incubation.

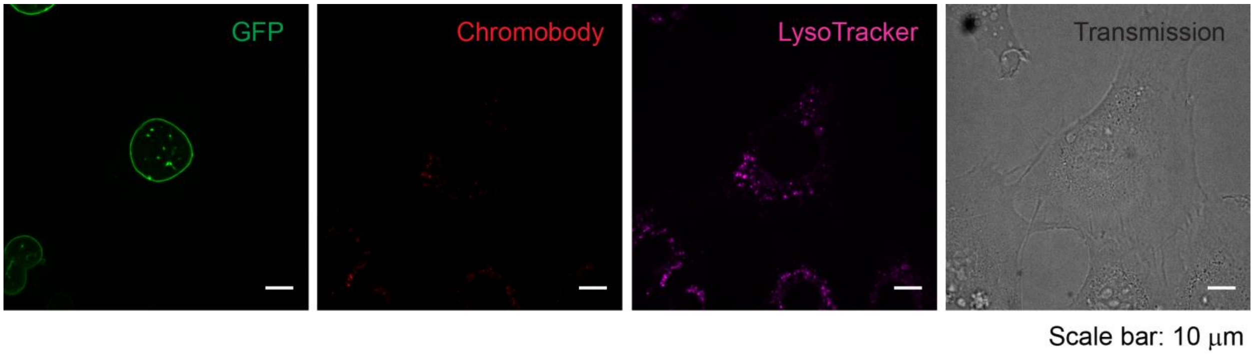

Supplementary Figure 5. Delivery of free chromobodies into MEF-G-LMNA cell. 25 nM free chromobodies was incubated with MEF-G-LMNA. 2 h after incubation, the residual free chromobodies in the medium were washed out by PBS, and the cells were incubated in Live Cell Imaging Medium for the following live cell imaging process. LysoTracker was used for lysosomes tracking during the imaging process.

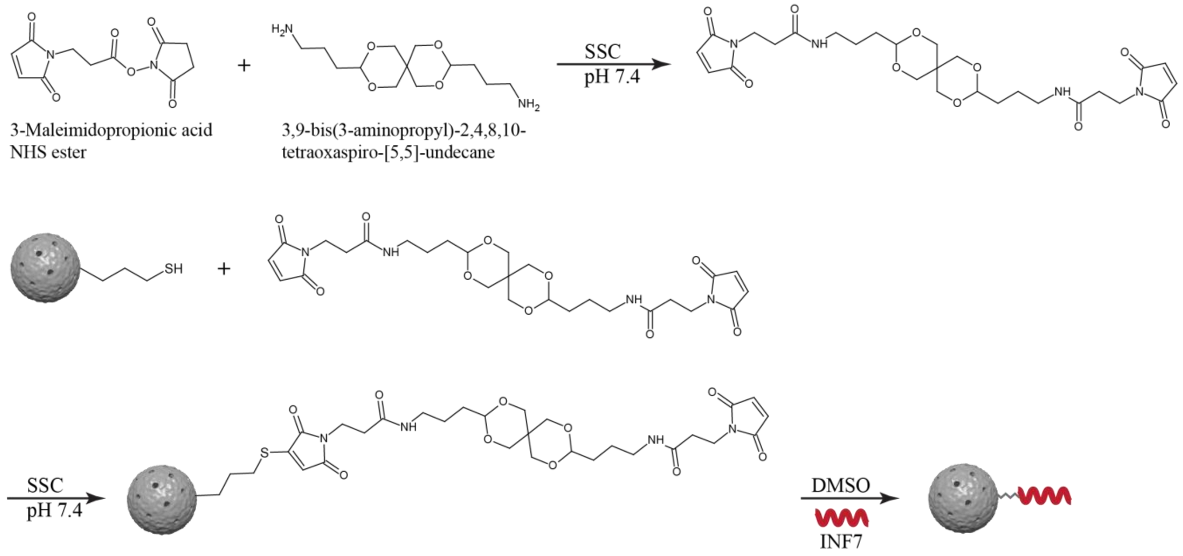

Supplementary Figure 6. Conjugation of INF7 peptide to MSNs *via* pH-responsive acetal linker. MSN-SH was conjugated with pH-responsive acetal linker (3,9-bis(3-aminopropyl)-2,4,8,10-tetraoxaspiro-[5,5]-undecane) (AK linker) *via* the maleimide-NHS heterobifunctional crosslinker (3-Maleimidopropionic acid NHS ester) yielding MSN-AK-linker. INF7 peptide consisting of cysteine on its C-terminus was covalently attached to MSN-AK-linker through the maleimide-thiol reaction.

Supplementary Information

The calculation of chromobody loading capacity in MSNs

- The radius of a MSN= $50\ nm$
- Particle volume (sphere) $= \frac{4}{3}\pi r^3 = 5.236 \times 10^5\ nm^3 = 5.236 \times 10^{-16}\ cm^3$
- Pore volume of MSNs (measured by N_2 sorption): $2.3\ cm^3/g$
- Pure silica density: $2.648\ g/cm^3$
- 1 g silica has: $\frac{1g}{2.648\frac{g}{cm^3}} = 0.38\ cm^3$
- 1 g MSN has: $0.38\ cm^3 + 2.3\ cm^3 = 2.68\ cm^3$
- MSN density assumption: $\frac{1g}{2.68\ cm^3} = 0.37\ g/cm^3$
- Single particle weight $= 5.236 \times 10^{-16}\ cm^3 \times 0.37\ g/cm^3 = 1.94 \times 10^{-16} g$
- **1 mg MSN has $\frac{10^{-3} g}{1.94\times10^{-16} g} = \mathbf{5 \times 10^{12}}$ particles**
- Molecular weight of chromobody: $14000\ g/mol$
- **Chromobody loading capacity: $\boldsymbol{70\ \mu g\ chromobody/mg\ MSN}$**
- $\boldsymbol{70\ \mu g\ chromobody} = \frac{70\times10^{-6} g}{14000\frac{g}{mol}} = 5 \times 10^{-9} mol$

  $$= 5 \times 10^{-9} mol \times 6.02 \times 10^{23}\ \frac{molecules}{mol} = \boldsymbol{3 \times 10^{15}\ chromobody\ molecules}$$

- **Chromobody loading capacity** $= \frac{3\times10^{15}\ chromobody\ molecules}{5\times10^{12}\ nanoparticles} = \frac{\boldsymbol{600\ chromobody\ molecules}}{\boldsymbol{MSN}}$

Supplementary Table

Sample	Element	mg[1]	mmol[1]	Molar ratio
	Si	563.3 ± 91.3	20.1	1
MSN-Ca^{2+}	S	10.07 ± 0.34	0.31	0.016
	Ca	0.29 ± 0.09	0.007	0.0004
	Si	476.8 ± 9.97	17.0	1
MSN-Ca-Cb	S	9.11 ± 1.15	0.28	0.017
	Ca	0.17 ± 0.03	0.004	0.0003
	Si	532.0 ± 47.73	19.0	1
MSN-Ni^{2+}	S	9.8 ± 0.32	0.31	0.016
	Ni	0.44 ± 0.01	0.007	0.0004
	Si	507.8 ± 4.45	18.1	1
MSN-Zn^{2+}	S	10.12 ± 0.55	0.32	0.017
	Zn	1.49 ± 0.03	0.023	0.0013

Notes: [1] The element amount is relative to 1 g of the measured sample.
[2] Triplicate experiments were performed in each sample.

Supplementary Table 1. Elemental analysis of MSN-M^{2+} and MSN-M-Cb samples via ICP-OES approach.
